# Proteome-wide analysis of *in situ* aged fibroblasts

**DOI:** 10.18632/oncotarget.3176

**Published:** 2015-01-13

**Authors:** Daniel M. Waldera-Lupa, Kai Stühler

**Affiliations:** Molecular Proteomics Laboratory, Institute for Molecular Medicine, Heinrich-Heine-University Düsseldorf, Germany. Biomedical Research Center (BMFZ), Heinrich-Heine-University Düsseldorf, Germany

Aging is a multifactorial process that is characterized by distinct molecular and biological changes [1]. However, deciphering the molecular and cellular mechanisms of aging *in vivo* is far from complete. It has been suggested that the aging process of the skin is mainly determined by alterations of its dermal stroma consisting predominantly of fibroblasts and the extracellular matrix [2, 3]. In this context the stromal-epithelial interactions by senescent fibroblasts is also discussed to contribute to age-related pathology, including cancer [3].

The homeostasis of the dermis is primarily based on cellular adaptation and damage clearance and thereby prone to age-related changes. As the major cell source in the dermis and a long-lived cell system, dermal fibroblasts are able to accumulate aging-associated alterations and adapt their cellular functions. A broadly applied fibroblast aging model is based on the limited replicative lifespan of these cells. Although senescence is one of the hallmarks of aging [1], it is unclear to which extent senescence induced *in vitro* is a representative model for cell and organ aging *in vivo*. Moreover, most studies analyzing aging of fibroblasts *in vivo* and *in vitro* focused on single genes or pathways, while comprehensive investigations of aging-associated alterations at the proteome-, transcriptome- and miRNAome -wide levels are sparse [4].

We analyzed an *ex vivo* model of *in situ* aged human dermal fibroblasts, obtained from 15 adult healthy donors from three different age groups using an unbiased quantitative proteome-wide approach applying label-free mass spectrometry [5]. We identified a total of 2409 proteins, including 43 proteins with a significant age-associated expression, whereof 20 and 23 proteins exhibit positive and negative correlation with age, respectively. Most of the differentially abundant proteins have not been described in the context of fibroblasts' aging before, but the deduced biological processes confirmed known hallmarks of aging [1]. Based on the integration of comprehensive data sets from expression studies at the protein [5], mRNA [6] and miRNA [7] levels, we presented a synoptic view of molecular changes associated with the aging of human dermal fibroblasts in the skin. We demonstrated that 47 % and 63 % of the proteins and transcripts, respectively identified in these cells exhibited a constant abundance across different donor age groups suggesting that *in situ* aged fibroblasts exhibit a moderate age-associated cellular phenotype [5]. A further observation supporting the moderate age-associated cellular phenotype was the low number of significantly changed mRNAs (137), proteins (43) and miRNAs (12). The fact that 77 % of the age-associated proteins were not linked to expression changes of the corresponding mRNA transcripts suggested that most of the age-associated alterations detected at the proteome level are likely caused by other processes, such as post-transcriptional regulation, translation efficiency, protein stability or modifications, rather than by differential regulation of gene expression.

Based on the deduced biological processes of the proteome-wide study our data are in good agreement with the concept of geroconversion, which is an irreversible process that is driven by mTOR-signaling and entails the loss of proliferative potential and the acquisition of cellular hallmarks of aging [8]. Age-associated alterations of the proteome, transcriptome and miRNAome of fibroblasts observed in our study, indicated an age-associated decline in proliferative capacity and age-associated features of geroconversion manifesting in the acquisition of several hallmarks of fibroblast aging [4] encompassing altered proteostasis, altered cell-cell signaling, hypertrophy and altered cell organization, altered stress response, altered RNA metabolism and translation.

Our study has several unique features. First, we used an *ex vivo* model of *in situ* aged human dermal fibroblasts which has been suggested to closer represent *in vivo* situation of aging than commonly used senescence-related *in vitro* aging models. In order to detect alterations associated with donor age and to minimize confounding aspects (i) the study was restricted to dermal fibroblasts from female donors to avoid sex-specific influences, (ii) all cells were studied at low passage numbers (passage 3) and (iii) were isolated from the same intrinsically aged skin area, thereby minimizing variances due to body location or different exposure to the external milieu. Second, our data was generated by an unbiased quantitative proteomics approach applying label-free mass spectrometry. Thereby, we identified and quantified more than 2400 proteins of different pathways at the same time. Applying this approach we were able to describe the intracellular proteins as an interacting network analyzing a variety of different pathways involved in the aging process instead of focusing on single genes or pathways. Third, using an integrative bioinformatical approach to combine proteome, transcriptome and miRNAome data we were able to draw a comprehensive picture of the molecular changes associated with the aging of human dermal fibroblasts in the skin.
